# Supplementary material for: Drosophila Larval Models of Invasive Tumorigenesis for In Vivo Studies on Tumour/Peripheral Host Tissue Interactions during Cancer Cachexia
Source: Int J Mol Sci. 2021 Aug 2;22(15):8317. doi: 10.3390/ijms22158317 (PMC8347517; doi:10.3390/ijms22158317)
Supplement: Supplementary file 1 [file ijms-22-08317-s001.zip › Supplementary Text.pdf]

***Drosophila* larval models of invasive tumourigenesis for in vivo studies on tumour/peripheral host tissue interactions during cancer cachexia.**

Supplementary Figure Legends

Figure S1. Representative images of imaginal disc tissues. (A,C,E-L) Immunofluorescent confocal images of larval imaginal discs stained with DAPI (blue) from a control (*w*^{1118}) animal **(A)** animal carrying hyperplastic (*rotund*>*Yki*^{S168A}) tumour **(C)**, non-invasive neoplastic tumour (*dlg*$^{40.2}$) **(E, F),** malignant neoplastic MARCM-generated eye tumours (*Ras*^{V12}, *scrib*^{1}) **(G, H)** or *hedgehog-gal4* overexpressing *Ras*^{V12}, *scrib*^{IR} (*Ras*^{V12}, *scrib*^{IR}) at 6 or 12 days AED. Imaginal discs from 6- or 12-days AED *e*^{1} larvae **(K, L). (B)** Brightfield image of *w*^{1118} adult fly at 12 days AED. **(D)** Brightfield image of dead *rotund*>*Yki*^{S168A} pupa at twelve days AED.

Figure S2. Representative images of animals and larval cuticles and skeletal muscle. (A,C,E-L) Immunofluorescent confocal images of body wall muscle from animals of genotypes and ages as indicated in Figure S1. **(B)** Brightfield image of a *w*^{1118} adult fly at 12 days AED. **(D)** Brightfield image of dead *rotund*>*Yki*^{S168A} pupa at twelve days AED.

Figure S3. Representative images of the 5th segment of the 7th ventral muscle. (A-F'', I-L'') Immunofluorescent confocal images of the 5th segment of the 7th ventral larval muscle (outlined in yellow in the grey scale images) from animals of genotypes an ages as indicated in Figure S1. Tissues were stained with Phalloidin (green) and LipidTOX (red and grey). **(G)** Brightfield image of *w*^{1118} adult fly at 12 days AED. **(H)** Brightfield image of dead *rotund*>*Yki*^{S168A} pupa at 12 days AED. The panels denoted with (') represent lipid staining alone. The panels denoted with (") represent a transversal orthographic view of the muscle shown in (A-F,I-L), with a 2X zoom. Areas demarked by red boxes represent zoomed in images in insets.

Figure S4. Impl2 does not mediate muscle wasting in larval models of tumour induced cachexia. (A) mRNA expression levels of *impl2* in cuticles and skeletal muscle from 7-day-old wild type larvae (*w*^{1118}) or larvae carrying *hedgehog-gal4* overexpressing *Ras*^{V12}, *scrib*^{IR} tumours without (*w*^{1118}) or with

impl2 knockdown (*impl2-IR)*. One-way ANOVA with Tukey post-hoc correction. Mean ± SEM is indicated. *p≤0.05; ***p≤0.001. **(B)** Quantification of the percentage of cuticle covered by muscle in 12-day-old larvae with *hedgehog-gal4* overexpressing Ras^{V12}, $scrib^{IR}$ tumours without (w^{1118}) or with *impl2* knockdown (*impl2-IR)*. T-test. Mean ± SEM is indicated. No statistical significance; p>0.05.

Supplementary Table Legends

Table S1: RNAseq data of cuticles and skeletal muscle from control and cachectic animals. Complete data set from RNAseq analysis of cuticles **and skeletal muscle** from control and cachectic animals (Figure 4). Control condition is labelled in blue and named 'cont', while the data corresponding to cachectic animals is labelled in red and named 'treat'.

Table S2: Fly lines and full genotypes of animals used throughout the study.

Table S3: Sequences of primer sets used throughout the study.
