# Supplementary figures and images for: Drosophila Larval Models of Invasive Tumorigenesis for In Vivo Studies on Tumour/Peripheral Host Tissue Interactions during Cancer Cachexia
Source: Int J Mol Sci. 2021 Aug 2;22(15):8317. doi: 10.3390/ijms22158317 (PMC8347517; doi:10.3390/ijms22158317)

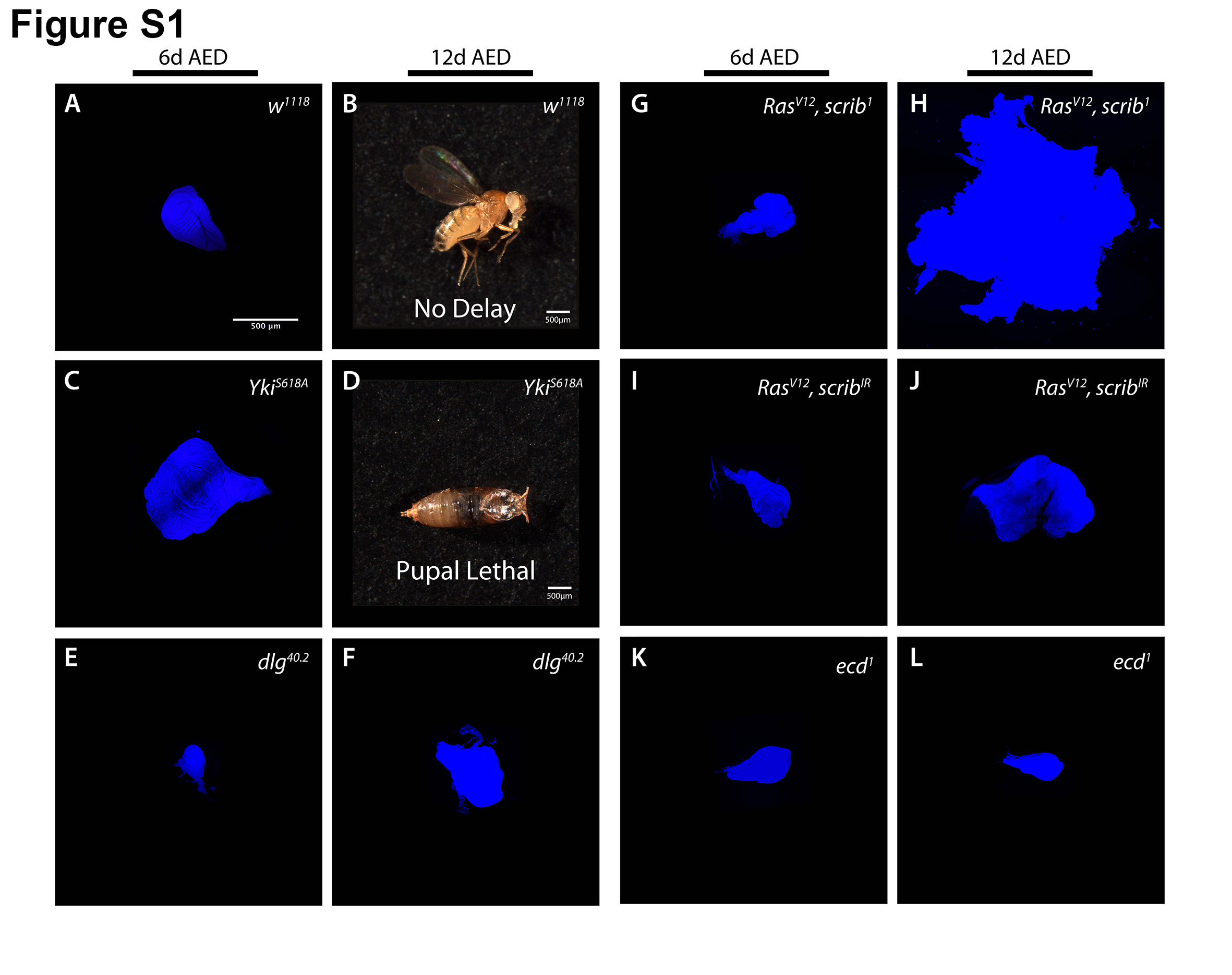

Supplement: Supplementary file 1 [file ijms-22-08317-s001.zip › Supp. Fig. S1.JPEG]

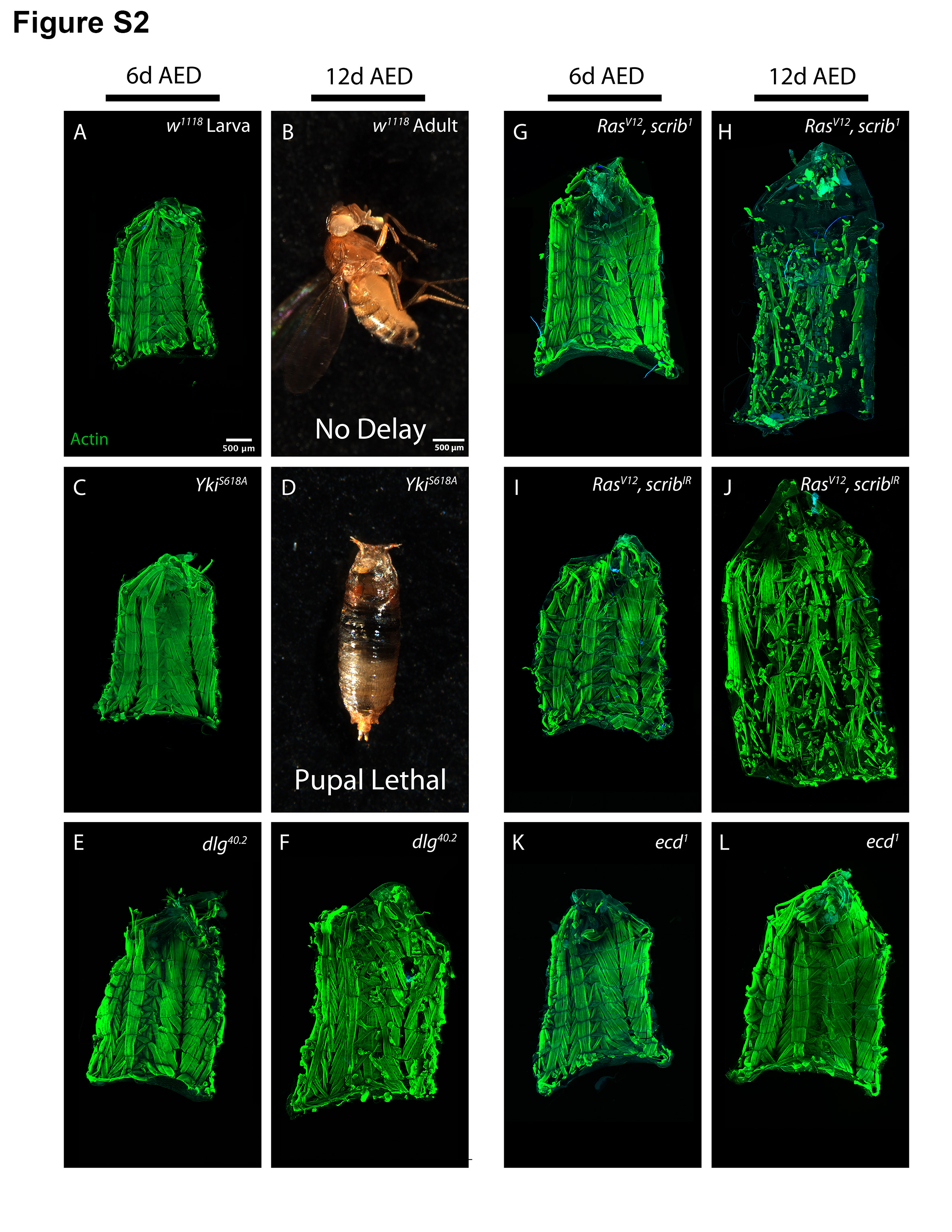

Supplement: Supplementary file 1 [file ijms-22-08317-s001.zip › Supp. Fig. S2.JPEG]

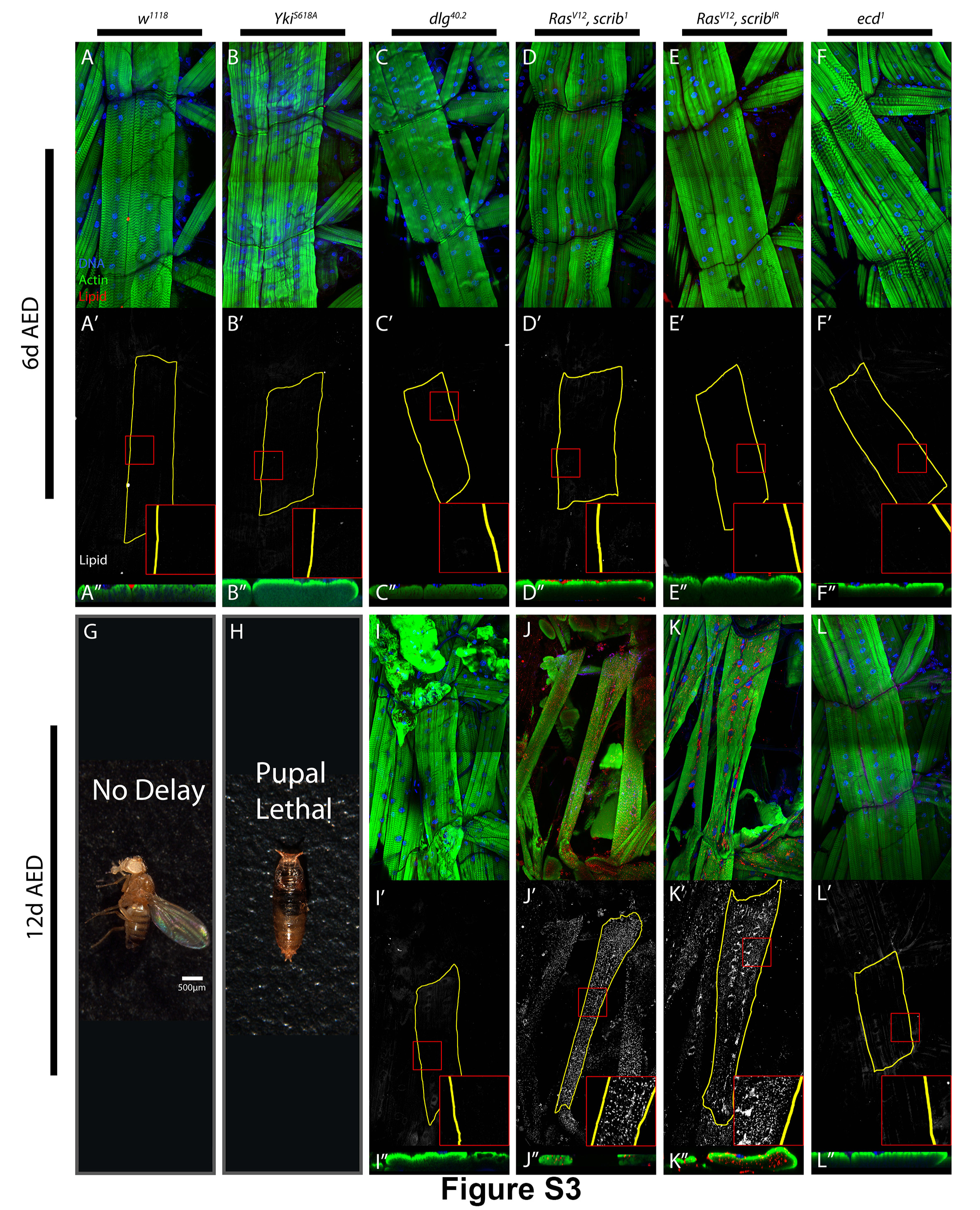

Supplement: Supplementary file 1 [file ijms-22-08317-s001.zip › Supp. Fig. S3.jpeg]

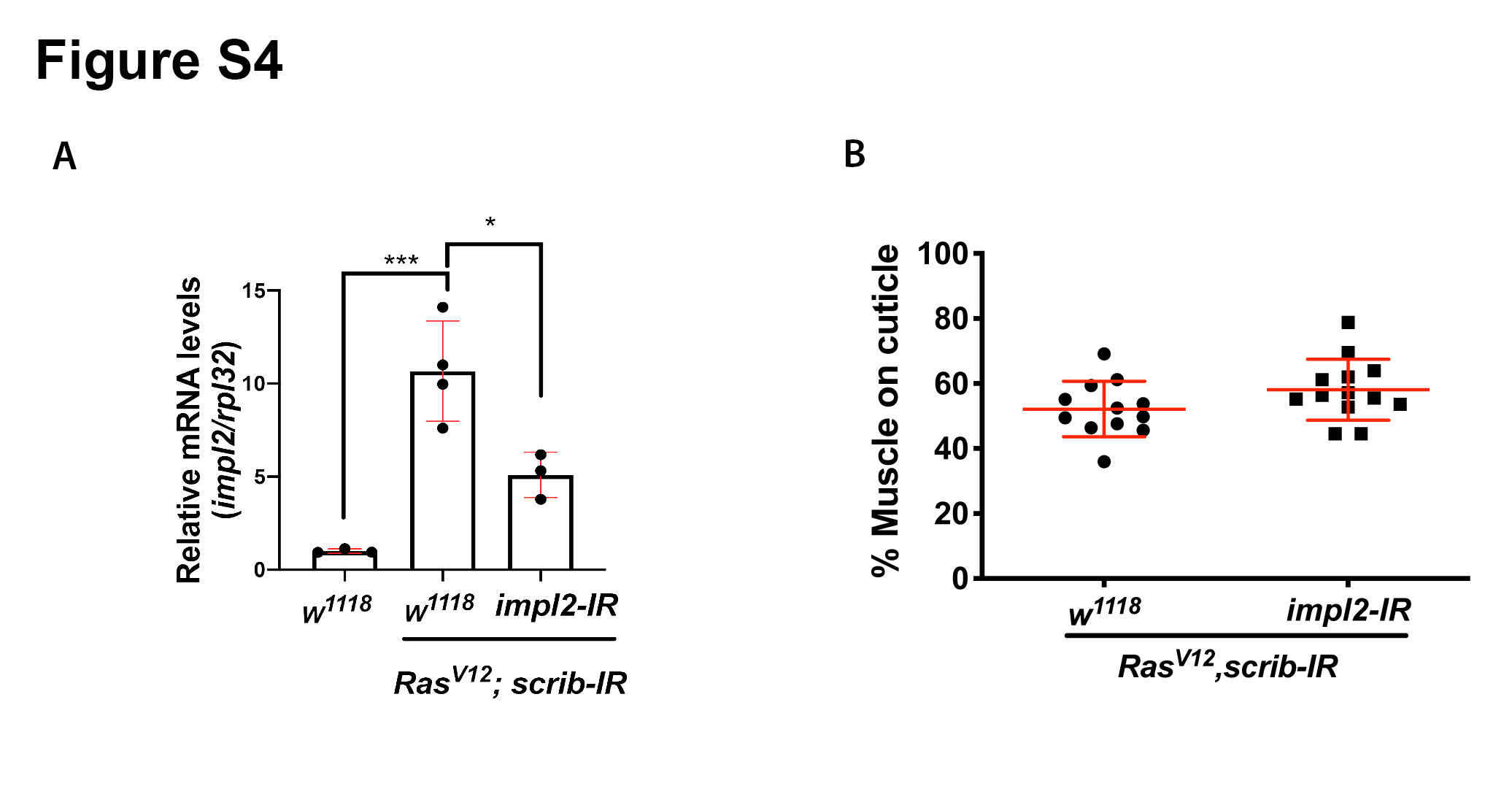

Supplement: Supplementary file 1 [file ijms-22-08317-s001.zip › Supp. Fig. S4.JPEG]
